# Weighted phase lag index stability as an artifact resistant measure to detect cognitive EEG activity during locomotion

**DOI:** 10.1186/1743-0003-9-47

**Published:** 2012-07-24

**Authors:** Troy M Lau, Joseph T Gwin, Kaleb G McDowell, Daniel P Ferris

**Affiliations:** 1 Human Neuromechanics Laboratory, School of Kinesiology, University of Michigan, Ann Arbor, MI 48109-2214, USA; 2 US Army Research Laboratory, Human Research and Engineering Directorate, Translational Neuroscience Branch, Aberdeen Proving Ground, Aberdeen, MD 21005, USA

**Keywords:** Electroencephalography (EEG), Walking, Movement artifact, Artifact removal, Connectivity, Phase lag

## Abstract

**Background:**

High-density electroencephalography (EEG) with active electrodes allows for monitoring of electrocortical dynamics during human walking but movement artifacts have the potential to dominate the signal. One potential method for recovering cognitive brain dynamics in the presence of gait-related artifact is the Weighted Phase Lag Index.

**Methods:**

We tested the ability of Weighted Phase Lag Index to recover event-related potentials during locomotion. Weighted Phase Lag Index is a functional connectivity measure that quantified how consistently 90° (or 270°) phase ‘lagging’ one EEG signal was compared to another. 248-channel EEG was recorded as eight subjects performed a visual oddball discrimination and response task during standing and walking (0.8 or 1.2 m/s) on a treadmill.

**Results:**

Applying Weighted Phase Lag Index across channels we were able to recover a p300-like cognitive response during walking. This response was similar to the classic amplitude-based p300 we also recovered during standing. We also showed that the Weighted Phase Lag Index detects more complex and variable activity patterns than traditional voltage-amplitude measures. This variability makes it challenging to compare brain activity over time and across subjects. In contrast, a statistical metric of the index’s variability, calculated over a moving time window, provided a more generalized measure of behavior. Weighted Phase Lag Index Stability returned a peak change of 1.8% + −0.5% from baseline for the walking case and 3.9% + −1.3% for the standing case.

**Conclusions:**

These findings suggest that both Weighted Phase Lag Index and Weighted Phase Lag Index Stability have potential for the on-line analysis of cognitive dynamics within EEG during human movement. The latter may be more useful from extracting general principles of neural behavior across subjects and conditions.

## Background

The ability to measure cognitive brain dynamics with electroencephalography (EEG) during real-world behaviors has historically been challenging for neuroscientists. One of the most fundamental and difficult aspects of this challenge is to parse EEG from electromyographic, electroocular, and movement artifacts that occur during movement [1-5]. Overcoming this challenge would help researchers understand the cognitive dynamics that occur during everyday life. It would also have applications in various neurotechnologies, such as monitoring neurological conditions, and would greatly contribute to the understanding of the control of human movement. Movement artifacts in EEG recorded during walking include movement of electrodes, loss of skin contact, muscles activation associated with head stabilization (electromyographic artifact) [6,7], and cable sway that leads to electronic interference. Other electrical artifacts in EEG occur due to muscle activation associated with jaw clenching and blinking, and movement of the eye (electroocular artifacts). These latter artifacts are not specific to movement tasks but can still make it difficult to separate out EEG related from true cognitive dynamics.

Two recent papers [8,9] have demonstrated the ability to record event-locked cognitive EEG activity during locomotion despite the presence of movement artifacts. In the first analysis [8], the authors introduced an artifact template technique to remove gait-locked EEG activity, and were able to recover an event-locked p300 response associated with an oddball discrimination task even during running. In the second analysis [9], the authors used independent component analysis (ICA) to separate EEG channel activity during walking into brain, muscle, eye, and movement artifact signals. Using an inverse modeling approach, the authors were able to determine the anatomical locations of independent sources of brain activity that collectively formed the scalp level p300 response. While both were effective at removing walking artifact they each had their limitations. The first technique requires a regular and predictable pattern of movement over which an artifact template can be time locked. Such conditions do not exist for most types of artifacts and require specialized recording equipment in the lab (i.e., motion capture cameras). The second technique (ICA) requires significant post-processing methods, which would not be useful for brain-machine interface devices in their current state. Neither of these approaches can be used in real-time.

One alternative approach for reducing EEG artifacts has been to use machine learning techniques such as a neural networks [10] to extract the important signal. Earlier methods of artifact reduction that focus on phase, instead of amplitude, include Mean Phase Coherence [11], Phase Lag Index [12], Phase Locking Value [13], and Imaginary Coherency [14]. Each of these techniques have naturally progressed from one another. They have emerged from our understanding that phased-based measures of functional connectivity can be useful in removing artifact signals that primarily lie in amplitude space. It was not until Stam introduced the most recent family of Phase Lag Index measures however, that the artifact removing benefit of removing phase and anti-phase locked signal was demonstrated. To our knowledge, none of these techniques have been applied to EEG recorded during walking, so it is not known how effective they would be in the presence of gross body movements.

The next metric in this progression that could be used for reducing movement artifacts in EEG is the Weighted Phase Lag Index (WPLI). WPLI was introduced recently [15]. It extends Stam’s PLI measure, by introducing a phase-difference weighting normalization. WPLI could negate the need for standard EEG pre-processing techniques like noisy channel removal, noisy epoch removal, or artifact-laden epoch removal by filtering out artifacts on-line. The major advantage of having an on-line method for artifact rejection is that it would allow for fast implementation for assessing cognitive dynamics [16-18] and could also be used for brain-computer interfaces [19] that worked in real time. This would facilitate neurotechnology development that could be deployed outside the laboratory.

To test the potential of WPLI for the on-line assessment of cognitive dynamics, we collected EEG data from healthy human subjects standing and walking while engaged in a visual oddball discrimination task. The visual oddball discrimination task is an extremely well-studied [20,21] paradigm involving a subject viewing presentations of two stimuli; a ‘standard’ that occurs often, and an ‘oddball’ that occurs infrequently. The presentation of this oddball is known to create an event-related potential (ERP) at about 300 milliseconds after presentation of the oddball (p300), as measured by EEG. We hypothesized that WLPI would allow us to recover a p300-like event-related potential from movement artifact contaminated EEG recorded during walking that manifested from the phase relationships among channels. We also investigated a measure of WPLI Stability (WPLIS) to generalize the WPLI measure, classifying periods of high or low stability/volatility. The purpose of using WPLIS was to account for the intra-subject and inter-trial specificity in the WPLI response. It allowed us to generalize the WPLI response and quantify the gross changes in WPLI dynamics.

## Methods

Eight healthy, right handed, volunteers, with no history of major lower limb injury and no known neurological or locomotor deficits completed this study (age range 20–31 years). All subjects provided written informed consent prior to the experiment. The University of Michigan Internal Review Board approved the protocol and we complied with all standards defined in the Declaration of Helsinki. More detailed accounts of the data collection methods can be found in previous publications [9].

Subjects stood and walked (0.8 m/s), on a treadmill while we recorded 248-channel electroencephalography at 512 Hz (ActiveTwo, BioSemi, Amsterdam, The Netherlands). Concurrently, standard (80%) and target (20%) stimuli (0° or 45° rotated black crosses on a white background, respectively) appeared on a monitor placed at eye level about 1 m in front of the subjects. Each stimulus was presented for 500 ms separated by intervals of uniform variation between 500 ms and 1500 ms. For each gait condition (standing and walking), subjects performed an experimental block where they pressed a handheld button whenever the target stimulus appeared (active) and a control block where they did not press a button (passive). Triggers were sent from the computer and the handheld button to time-lock the presentation and reaction to the EEG data. Each data collection session began with the standing condition, followed by the walking condition. The standing block lasted 5 minutes each while the walking lasted 10 minutes. Subjects performed only a single block of each condition to minimize the effects of stimulus habituation.

### EEG processing for comparative analysis

All processing and analysis was performed in Matlab (The Mathworks, Natick, MA) using scripts based on EEGLAB (sccn.ucsd.edu/eeglab), an open source environment for processing electrophysiological data [22], as well as specialized code for this study.

For the majority of the analyses in this paper, all EEG channels were used. However, to compare the inclusion/exclusion of noisy channels, we identified noisy channels as in [8,23]. Data was initially high-pass filtered above 1 Hz. EEG noise removal parameters were then selected according the established standards of EEGLAB and it developers at the Swartz Center for Computational Neuroscience (University of California San Diego). EEG signals exhibiting substantial noise throughout the standing and walking conditions were removed from the data in the flowing manner: 1) channels with std. dev. > 1000 μV were removed, 2) any channel whose kurtosis was more than 5 std. dev. from the mean was removed, and 3) channels that were uncorrelated (r < 0.4) with nearby channels for more than 1% of the time-samples were removed. An average of 130.4 EEG channels remained after the exclusion of these channels (range : 89–164, stdev : 24.6). Channels were then re-referenced to an average of the remaining channels.

### Weighted Phase Lag Index

After collection, EEG signals were band passed around 4 ± 2 Hz using a 2^nd^ order Butterworth filter. Additional frequency bands around 6, 8, and 10 Hz were also investigated, though the 4 Hz band showed the most robust result. In future studies it may be useful to thoroughly explore the range of frequency bands, the impact of different bandwidths, and the cross communication between frequencies.

We calculated WPLI as explained by Vinck et al. [15]. Data were down sampled from 512 Hz to 51.2 Hz before calculation of instantaneous phase because of computational limitations. Interpolation was not used as it may have an effect on the calculation of phase. Because of the frequency band investigated we do not believe this downsampling significantly affected the metric. The instantaneous phase *φ*_*n,t*_, of each channel, for every time sample, was calculated by first taking the Hilbert transform of *Ψ*. *Ψ* is the matrix of n = 1…N channels (rows) and t = 1…T time samples (columns),

$${\tilde{\psi }}_{n,t}=H(\psi_{n,t})$$

and then computing the instantaneous phase,

$$\varphi_{n,t}=\tan^{-1}\left(\frac{{\tilde{\psi }}_{n,t}}{\psi_{n,t}}\right)\text{.}$$

Next, the phase difference between every channel pair was computed, for each time sample,

$$\Delta \Phi_{n_{i},n_{j},t}=\varphi_{n_{i},t}-\varphi_{n_{j},t}\text{.}$$

WPLI was calculated over ~488 ms sliding windows, with ~244 ms of overlap, via the following equation:

$$WPLI_{n_{i},n_{j},t}=\left|<\frac{\left|\sin(\Delta \Phi_{n_{i},n_{j},\tau })\right|}{\sin(\Delta \Phi_{n_{i},n_{j},\tau )}}>\right|$$

where ΔΦ*n*_1_, *n*_2,τ_ is a vector of phase differences spanning ~488 ms (25 time samples) and τ is the sliding time window index. At this point, since WPLI is an undirected measure, the total number of connections can be reduced from N^2^ to N(N-1)/2. For simplicity however we keep N^2^ connections here.

The purpose of the WPLI is to remove amplitude and phase synchronous based artifacts that are intrinsically mixed with brain activity. By operating in phase space, and maximally weighting ±90 degree phase differences, all signals associated with temporally acute, as well as uniformly driven sources, are omitted. Only phase lagging interactions, like those from a complex coupled oscillator system (e.g., the brain), are detected.

### Weighted Phase Lag Index Stability

We used the coefficient of variation, calculated over a 0.5 s sliding time-window, to calculate WPLI Stability. For a given WPLI window, *τ*, WPLIS was:

$$WPLIS_{n_{i},n_{j},\tau }=\frac{STD(WPLI_{n_{i},n_{j},\tau -0.5s}\cdots WPLI_{n_{i},n_{j},\tau })}{\langle WPLI_{n_{1},n_{2},\tau -0.5s}\cdots WPLI_{n_{i},n_{j},\tau }\rangle }$$

where STD is the standard deviation. This measure of temporal stability identifies periods of high/low WPLI variability. If WPLI is a measure of the functional flow of information between two brain regions (mapped to EEG channels in this case) [15], then a low WPLIS reflects continuous and uniform flow of this information. Conversely, if WPLIS is high then WPLI is highly irregular and an unstable dynamic information flow is implied.

### Weighted Phase Lag Index Stability Statistics

The error for all WPLIS was calculated as the standard error across all eight subjects. WPLIS changes were calculated in reference to the baseline by averaging the 0.5 s epoch prior to oddball presentation. Then, the minimum WPLIS value was extracted for the 1.5 s epoch following the oddball presentation. The error for that minimum value was then used to calculate z-scores and p-values for the minimum.

### Principal Component Analysis of WPLIS Event-Locked Response

We performed a principle components analysis (PCA), using MATLAB’s built in PCA function, on the channel pairs to achieve two goals. First, we wanted to extract the primary underlying event-locked response in the WPLIS brain network. Second, we sought to map the spatial distribution of the channel pairs that contributed most highly to the event-locked WPLIS response.

We generated a topographic map showing the extent to which each channel pair contributed to the first principal component (PC) of the WPLIS response. This was done by summing, for each channel of a channel pair, the positive contributions (PC loading) to the first PC. Channels that contributed most strongly and most often (as a part of various channel pairs) to the first PC, had the highest cumulative loading and the warmest topographic map color.

$$Cumulative\;PC\;Loading_{n_{i}}=\sum_{n_{j}=1}^{N}\{\begin{array}{cc} PC\;Loading_{n_{i}n_{j}}, & PC\;Loading\geq 0\\ 0, & PC\;Loading<0 \end{array}$$

## Results

**Figure 1:**
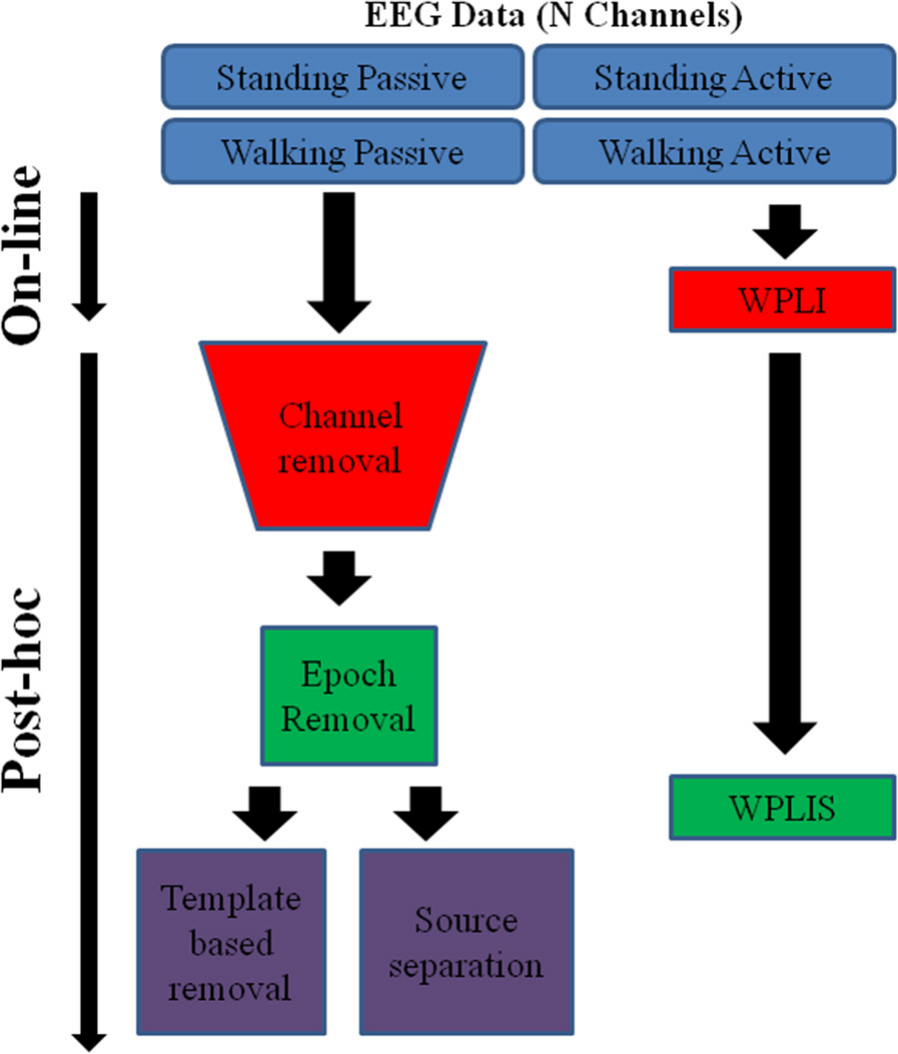
**Flow chart representing the four experimental conditions and the two EEG processing streams that have been used for EEG recorded during human locomotion.** The top block shows the four conditions over which EEG was recorded; standing while passively engaged in the oddball task, standing while actively engaged in the oddball task, walking while passively engaged in the oddball task, and walking while actively engaged in the oddball task. WPLI can be calculated entirely on-line while most other processing techniques must be calculated offline.

The conventional approach to analyzing stimulus-locked EEG signals with high artifact requires significant post-processing and cannot be done on-line. Figure 1 summarizes this paper. It demonstrates the four conditions in this EEG study along with the conventional (left) and WPLI (right) processing methods. Notice the time arrows along the left side demonstrating which methods can be computed on-line and which must be done post-hoc.

**Figure 2:**
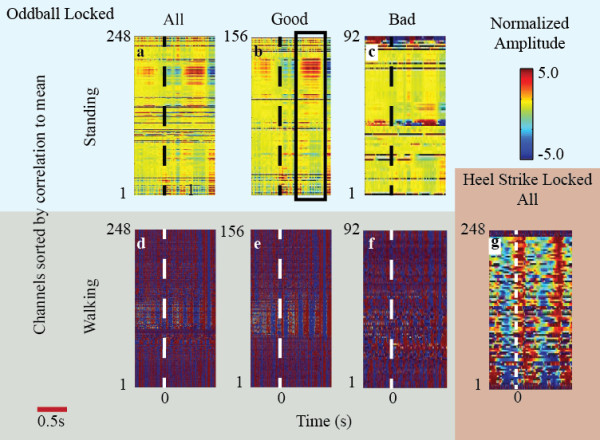
**Oddball and heel-strike locked voltage amplitude responses for a single subject (1) plotted over epochs from 0.5 s before to 1 s after the time-locking event.** The color mapped oddball, time-locked, voltage responses of all (**a**, **d**), clean/good (**b**, **e**), and bad (**c**, **f**) channels during standing (top row) and walking (bottom row) are shown. Clean channels are defined as those free of movement and EEG artifact, and bad channels are those omitted per the criteria discussed in methods. The standing cases show the cognitive dynamics that can be resolved time-locked to the oddball. The walking cases demonstrate that these dynamics are lost due to walking artifact. Panel (**g**) shows the voltage amplitude response locked to left heel-strike and how significant the walking artifact is. Each channel voltage color is normalized to its average voltage. Channels are sorted and numbered by their correlation to the mean voltage signal with higher correlated signals at the bottom. The black box around 300 ms in (**b**) shows a example component of the p300 negative deflection across clean channels. This deflection is not visible during walking (panel **e**).

Figure 2 shows the EEG signals during standing and walking time-locked to the onset of the visual oddball stimulus (Figure 2). We plotted the average scaled and normalized voltage response, for a single subject, from 0.5 s before stimulus onset to 1.0 s after stimulus onset for all channels (1a, 1d), non-noisy (good) channels (1b,1e), and noisy (bad) channels (1c,1f) as color-coded horizontal lines. Some noisy channels appeared clean for the standing cases because the noisy channels were defined across the standing and walking conditions, as well as across time periods not within these epochs. For the standing case, a p300 response (negative scalp potential deflection around 300–700 ms after oddball presentation, vertical black dashed line) was visible (black rectangle) and was more pronounced when noisy channels were omitted (Figure 2b). For the walking condition (Figures 2d-1f), no p300 cognitive potential was visible above the background noise. Figure 2g shows the EEG channel presented as locked to heel strike rather than oddball presentation. The artifacts from this event are many orders of magnitude larger than the voltage changes due to a cognitive response.

**Figure 3:**
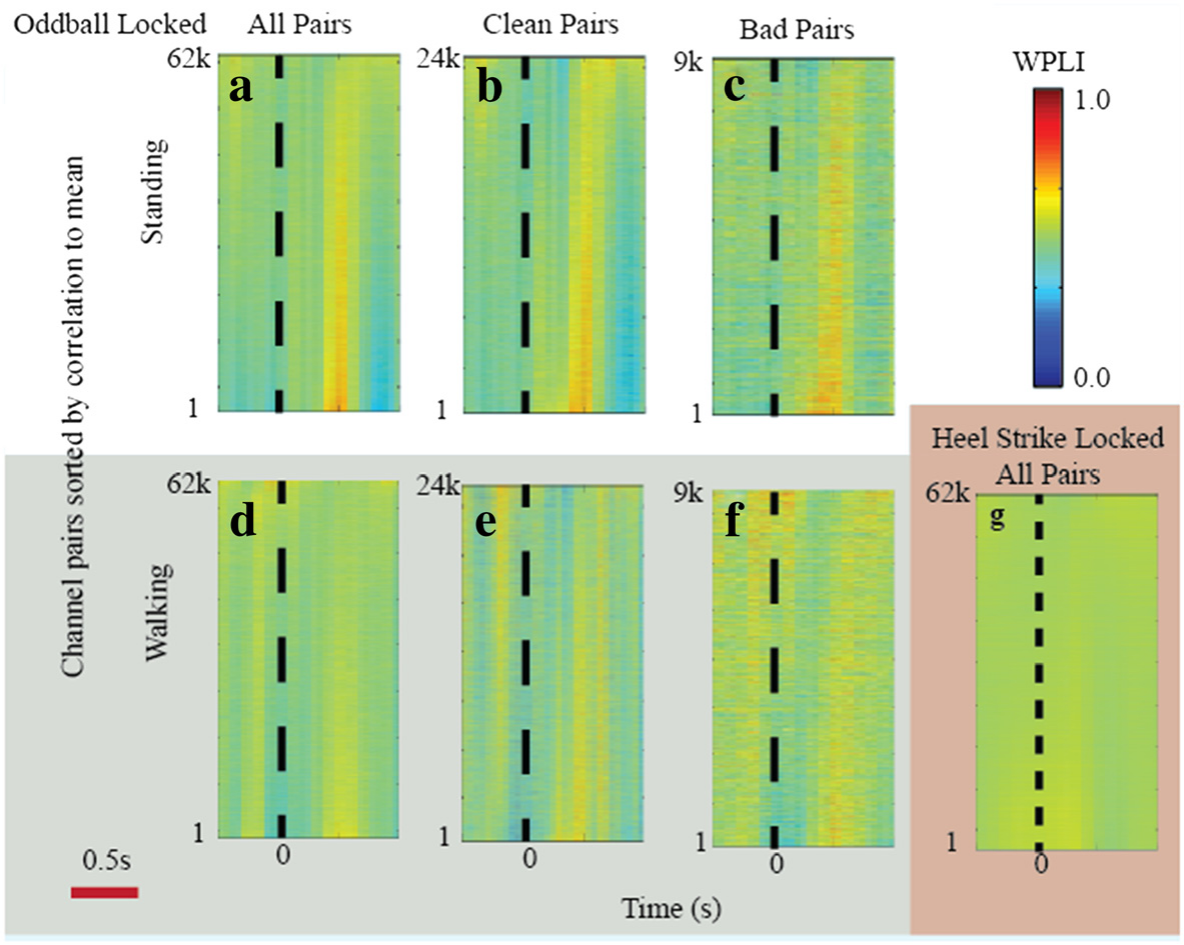
**Oddball and heel-strike locked WPLI deflections for the same subject as in Figure**2** plotted over epochs from 0.5 s before to 1 s after the time-locking event.** The color mapped WPLI response of all (**a**, **d**), clean (**b**, **e**), and bad(**c**, **f**) channel pairs (62 k = 61,504) during standing (top row) and walking (bottom row) are shown. Only data from the first 25% of each session was used. Channel pair WPLI measures are sorted and numbered by their correlation to the mean WPLI response. There is a clear and uniform WPLI deflection across almost all channels. This deflection can be seen even when both channels are considered bad and for the walking case. Panel (i) shows the WPLI deflections time-locked to left heel-strikes.

The WPLI results demonstrate a much clearer p300-like deflection for both standing and walking. Figure 3 shows WPLI results for the same subject, session, and epoch data as in Figure 2. The WPLI values are plotted for each channel pair (instead of for each channel as in Figure 2) because WPLI is a network approach. Figure 3 shows that a robust event-locked WPLI deflection exists, even across electrode pairs containing previously defined noisy channels (Figure 3c). Even in the walking case (Figure 3d-f), where the voltage amplitude signal was dominated by artifact, a clear event-locked cognitive response is visible. Figure 3g demonstrates the effectiveness of WPLI in removing movement artifact as the previously strong heel-strike artifacts shown in Figure 2 are mostly removed.

**Figure 4:**
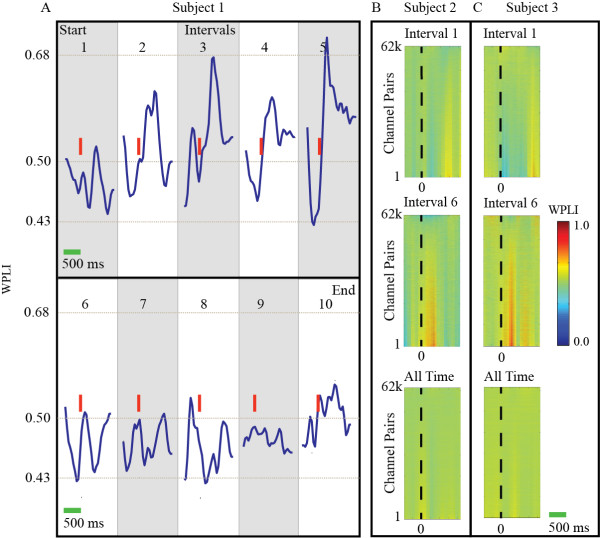
**Variability of time and subject of WPLI response to oddball stimulus. A**) WPLI averages taken over 10 distinct 30 s intervals to highlight the time varying nature of the WPLI event-locked response. The first 30 s of the trial were averaged in interval 1 and the last 30 seconds in interval 10. Red vertical lines indicate the onset of the oddball stimulus in each interval. Subject 1 is the same subject used in previous figures. **B**,**C**) Channel pair WPLI responses for selected intervals and over all time demonstrating the variability of WPLI responses across subjects.

The WPLI results were not consistent across time or subjects. Figure 3 is limited to the first two minutes (~25%) of the trial for the subject. The same subject’s WPLI response, when viewed across the whole time period as shown in Figure 4a. It is highly variable and does not provide a consistent response. In addition, the timings of distinct WPLI deflections, were variable across subjects (Figure 4b,c). This suggests the WPLI may not be the ideal measure for making generalizations of cognitive dynamics with consistency between subjects and over time.

**Figure 5:**
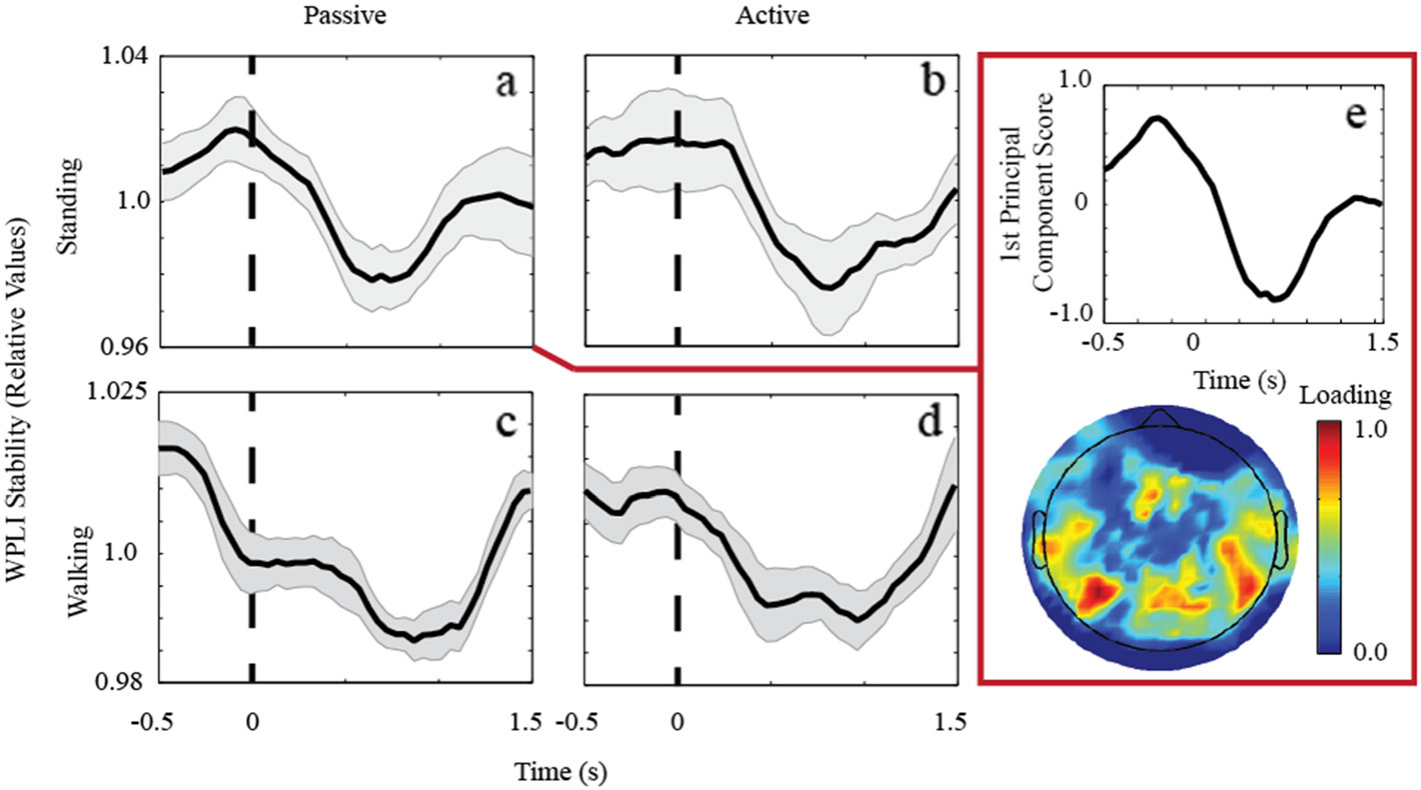
**The average WPLIS response across subjects for the standing/passive (a), standing/active (b), walking/passive (c), and walking/active (d) conditions.** Each case shows a statistically significant WPLIS decrease that follows the oddball presentation with a downwark peak at ~600 ms. For the active cases (b, d) the negative deflections are more pronounced. For the passive standing case a 3.6% + −0.8% change from baseline was measured (p = 0.0001) while for the active standing case a 3.9% + −1.3% change from baseline was measured (p = 0.0028). For the passive walking case a 2.4% + −0.3% change from baseline was measured (p = 0.0001) while for the active walking case a 1.8% + −0.5% change from baseline was measured (p = 0.0001). The EEG electrodes most strongly contributing to the 1^st^ principal component are shown in panel **e**. As expected strong visual cortex connectivity is present as the subject(s) observe the computer screen.

WPLIS provided a consistent and robust measure of oddball presentation (Figure 5). The grand mean WPLIS (averaged over all channel pairs and subjects within a given session type), time-locked to the oddball stimulus, is shown in Figure 5. The shaded regions indicate the standard errors across subjects. The sliding 0.5 s window used to calculate WPLIS smoothes the deflections seen in WPLI (Figure 3), therefore a longer (−0.5 s through 1.5 s) epoch is shown in Figures 4 and 5. For the standing and walking conditions (Figures 5), WPLI stabilization (i.e. a WPLIS decrease) was observed across nearly all of the channel pairs. This statistically significant decrease began 300 ms after stimulus onset, peaked around 700 ms, and continued until approximately 1 s after the presentation of the oddball stimulus. For the passive standing case a 3.6% + −0.8% change from baseline was measured (p = 0.0001) while for the active standing case a 3.9% + −1.3% change from baseline was measured (p = 0.0028). For the passive walking case a 2.4% + −0.3% change from baseline was measured (p = 0.0001) while for the active walking case a 1.8% + −0.5% change from baseline was measured (p = 0.0001). Figure 5 also shows which electrodes, belonging to pairs, contribute to the 1^st^ principal component of activity. We see the strongest contribution for the posterior regions near the visual cortex.

**Figure 6:**
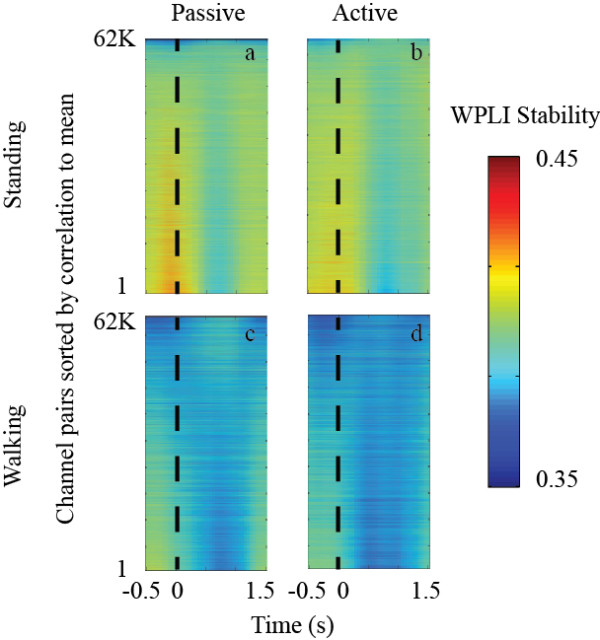
**Event locked WPLIS responses for the standing/passive (a), standing/active (b), walking/passive (c), and walking/active (d) conditions showinging pair-pair changes that are locked to the oddball stimulus.** Changes in the baseline level of WPLIS occur between standing and walking however the event-locked decrease is observable in both cases. Channel-pair WPLIS responses were averaged across subjects and then sorted by correlation to the mean.

Figure 6 shows the average WPLIS for each channel pair, time locked to the oddball stimulus, for each session type: standing/passive, standing/active, walking/passive, and walking/active. Channel pairs were sorted by correlation to the mean. In both the standing and walking conditions, a decrease in WPLIS occurred around 700 ms for the channel pairs. This response only occurred in fewer channel pairs during walking compared to standing.

## Discussion

Conventional approaches to EEG processing in high-artifact studies rely on post-processing that includes the removal of entire channels and time epochs that are laden with noise. After these steps are taken they often require large averages over trials and subjects to negate the remaining artifact effects. After removal of noisy channels, the remaining noise that was not time-locked to the cognitive event of interest is assumed to be smoothed and removed by the averaging of voltage traces over many trials when calculating event-related potential (ERP) plots. This approach can capture p300-related deflections under controlled artifact-limited conditions.

WPLI, a dynamic network functional connectivity measure, was less sensitive to gait-phase locked artifacts than conventional EEG channel voltage analyses. However, WPLI responses that were time locked to the appearance of a visual oddball stimulus during walking were highly variable across subjects and trials. We found that a stability measure of WPLI (i.e. WPLIS) provided a more robust p300-like cognitive event during walking. In the most general sense, a stability measure of any metric will provide a more generalized measure of its dynamic properties. The stability measure used here is analogous to the Fano Factor [24], which has been expanded to generalize the inter-spike intervals of neuronal firing. WPLIS allows for the comparison and averaging of responses within sessions and between subjects.

Our results suggest that WPLI provides a more useful technique for on-line analysis of cognitive dynamics during human walking than WPLI alone. However these results suggest that WPLI measures a more complex and specific activity than conventional amplitude-based measures. This leads to significant intra- and inter-subject variability and simple averaging of WPLI fails. WPLIS can be used for grouped analyses of WPLI across subjects and different studies. The findings are an important step toward developing a computational methodology to analyze EEG activity while humans interact in a real-world environment.

In another recent study [8], the researchers used an event related template to remove stride-synchronous movement artifacts. Gwin et al. [8] collected both EEG and kinematic data, created an artifact template by first time warping stride-locked EEG signals to uniform lengths in time, then averaged them. This template was subtracted from the EEG activity and the signal was unwarped, leaving only EEG activity and artifact that was not concurrent with each stride. While this method was useful in removing walking artifact, it is limited in its applicability to controlled situations, uncommon in real-world environments. It also does not account for random and unpredictable movement artifacts that may otherwise obscure the cognitive signal. Furthermore, by removing all EEG signal concurrent with stride, possible brain dynamics linked to stride were also removed [23]. The WPLIS measure does not require such an *a priori* knowledge of the nature of the artifact, nor the inclusion of kinematic movement data. Another major advantage of the WPLIS measure over the event related template technique is that WPLIS detection of cognitive processing can be conducted in an on-line fashion. The event related template technique requires considerable post-processing after data collection [8], the inclusion of the entire data set, and significant human analyzer input. Thus, although both techniques have demonstrated success as decoding electrocortical events related to cognitive dynamics, there are clear reasons for choosing one over the other depending on the situation of the data collection.

The WPLI and WPLIS measures are both network-based approaches to quantifying electrocortical dynamics. Several network-based approaches for understanding static and dynamic brain activity have gained considerable acceptance in recent years [25-29]. Network-based approaches allow for a broader parameter space (N^2^ as opposed to N, where N is the number of EEG channels) in quantifying brain activity. In addition, network-based approaches are driven by the *interactions* between sources of activity, instead of the individual sources themselves. This allows for a more complex characterization of activity. Network approaches recover the functional connectivity of the active brain [25,26]. Specifically, functional connectivity is the dynamic *measured* connectivity that reflects the anatomical connectivity and the underlying processes occurring in the brain at a given time. WPLI, in particular, is a functional connectivity measure that was designed to ignore non-brain sources of activity. The fundamental assumption is that stable, 90 degree out-of-phase, signals can only consistently arise from highly complex coupled harmonic oscillator systems (i.e., the brain) and not from external noise and artifact sources.

There are a number of areas where this methodology can be expanded to provide additional insight into underlying cognitive activity. Most importantly, the time-varying nature of WPLI dynamics should be studied in more detail. Figure 4 exhibits a complex and variable WPLI response across subjects and time. While this result may not be ideal for the consolidation of results, it does not imply that WPLI itself is inherently flawed. On the contrary, it suggests a deeper and more robust measure of cognitive dynamics that may vary across subjects and time. Further examination is needed to elucidate the complex WPLI response, much like initial work in the p300 voltage-amplitude response has led to a deeper understanding of the p3a and p3b [21,30].

The existence of several underlying processes contributing to the WPLI response described here would not be entirely surprising because WPLI is a functional connectivity metric. WPLI measures the interaction between channel pairs and quantifies the dynamic interaction between them, reflecting the functional connectivity between brain regions. In addition, the application of measures such as mean path length, clustering coefficients, and betweeness centrality could help researchers further understand connectivity changes within and between tasks [28,31,32]. Lastly, a comparison to the debiased WPLI may advance the results presented here [15].

We did not attempt to specifically describe the nature of changes in the WPLI response over the duration of the trials. Instead, we used WPLIS to identify periods of strong WPLI fluctuation, or conversely, WPLI steadying. WPLIS is not a direct measure of network communication, as WPLI is; instead, it measures *changes* in communication or functional network connectivity. A WPLIS decrease, indicating stabilization, reflects smaller changes in WPLI over time, meaning a more homogenous and temporally uniform processing state. Conversely, a WPLIS increase indicates larger changes in WPLI over time, and a transitioning processing state. The implementation of WPLIS was a necessary first step, but, as Figure 4 indicates, the dynamics of WPLI likely reflect functional connectivity which may be more complex than traditional voltage-amplitude measures.

## Conclusion

We demonstrated that WPLIS can be used to recover event-locked cognitive activity from artifact-contaminated EEG recorded during a walking task. WPLI had more limitations as a measure for long-term data collections and across subjects than WPLIS because of its sensitivity to a robust and yet unexplored range of brain connectivity dynamics. This work represents a logical step towards implementation of EEG-based brain imaging in real-world settings with on-line artifact removal.

## Abbreviations

WPLI: Weighted phase lag index; WPLIS: Weighted phase lag index stability; EEG: Electroencephalography; ERP: Event related potential.

## Competing interests

There are no competing interests in this work.

## Authors’ contributions

TL wrote the programming and performed all the analyses presented in this study. JG performed the experiments, collecting data on all subjects in this study. KM provided the inspiration for the use of WPLI and provided useful feedback at all stages. DF provided the inspiration for the experiment and provided useful feedback at all stages. All authors contributed to the writing of the manuscript and approved the final version.
